# Do older manual workers benefit in vitality after retirement? Findings from a 3-year follow-up panel study

**DOI:** 10.1007/s10433-020-00590-7

**Published:** 2020-11-04

**Authors:** Anushiya Vanajan, Ute Bültmann, Kène Henkens

**Affiliations:** 1 Netherlands Interdisciplinary Demographic Institute, Lange Houtstraat 19, 2511 CV The Hague, The Netherlands; 2 Department of Health Sciences, Community and Occupational Medicine, University Medical Center Groningen, University of Groningen, Broerstraat 5, 9712 CP Groningen, The Netherlands; 3 Faculty of Social and Behavioural Sciences, University of Amsterdam, Nieuwe Achtergracht 129-B, 1018 WT Amsterdam, The Netherlands

**Keywords:** Physical labour, Health, Well-being, Liveliness

## Abstract

Vitality is the feeling of physical and mental aliveness. Vitality benefits individual, organizational and societal well-being. However, we know much less about the dynamics in the levels of vitality and its’ precursors. This study investigates the effects of retirement on vitality and how this effect differs between manual and non-manual workers and by baseline levels of vitality. We used two waves of the NIDI Pension Panel Survey, collected in the Netherlands in 2015 and 2018. Data from 4156 older workers (*N* = 4156), of whom 1934 (46.5%) retired between waves, were analysed. Vitality is assessed in three ways, as: (1) a composite measure of vitality, and its subcomponents (2) energy and (3) fatigue. Conditional Change OLS Regression models demonstrated that retirement is associated with improved vitality and decreased fatigue. Older workers who retire from manual work at wave 1 experienced the largest gains in vitality and highest declines in fatigue at wave 2, compared to those who remained employed. Retirement was more advantageous for older workers who experienced poor vitality and high fatigue at wave 1. No such effects were found for energy. Older workers in manual work, those experiencing low vitality and high fatigue at wave 1, may benefit most from early retirement. Since opportunities for early retirement are highly restrictive, it is essential to provide these groups of workers with effective work accommodations and interventions that may not only improve their vitality and quality of working life, but also extend their participation in the labour market.

**Electronic supplementary material:**

The online version of this article (10.1007/s10433-020-00590-7) contains supplementary material, which is available to authorized users.

## Introduction

Retirement is a major life transition that has substantial effects on health (Van Solinge 2007). Many studies have looked at the effect of retirement on health, with conflicting and inconsistent results (Butterworth et al. 2006; van der Heide et al. 2013). These studies have examined the effect of retirement on a broad array of health measures, ranging from general self-rated physical and mental health to specific chronic health conditions. Rarely have these studies sought to understand the effect of retirement on vitality—the feeling of aliveness, both in the physical (healthy, capable and energetic) and mental (meaning and purpose) sense (Hennekam 2016).

Being vital is beneficial for individuals, organizations and society. A study among Dutch adults found vitality to be positively associated with increased economic, societal and social participation and negatively associated with societal costs (van Steenbergen et al. 2016), revealing the potential benefits of improving vitality on an individual level for societal well-being. Vital employees are described to be full of positive energy and mentally and physically strong (Kark and Carmeli 2009). They are also productive (Carmeli 2009) and are satisfied and successful at their jobs (Hennekam 2016; van Scheppingen et al. 2014). In contrast, low levels of vitality have been associated with burnout symptoms, especially with emotional exhaustion (Aleksandra Basinska et al. 2014). Further, low vitality has been shown to moderate the relationship between burnout and turnover intentions at work (Elçi et al. 2018). It has also been shown that older workers experiencing chronic health conditions prefer to retire early due to the poor vitality they experience in their daily lives (Vanajan et al. 2020). Currently, the labour market in the western world is experiencing an increase in public pension age and an abolishment of early work exit routes. In this context, it is crucial to better understand how vitality may change from work to retirement in order to sustainably extend working lives. Therefore, this study focuses on understanding the effect of retirement on older workers’ and retirees’ vitality. Vitality is measured as a combination of the positive state—energy, and the negative state—fatigue (Deng et al. 2015). To gain insights into how retirement affects vitality, this study will separately analyse the effect of retirement on vitality, and its subcomponents, energy and fatigue. By separately analysing vitality, energy and fatigue, we aim to distinguish which of these constructs should be addressed using governmental or workplace interventions and accommodations.

While numerous studies discuss the health consequences of retirement, more recent literature has emphasized the importance of understanding the heterogeneity in the health consequences of retirement (Henning et al. 2016). Some groups of retirees may find retirement beneficial to health as retirement could relieve them from the daily burden of work and give them more time to focus on their health, family and leisure activities. Others may find retirement detrimental to health as it may signify a loss in social relationships, daily routine and the sense of identity and purpose. These reactions to retirement might greatly depend on situational factors, such as the characteristics of the job one retires from, and personal characteristics, such as one’s health before retirement (Henning et al. 2016). This study aims to understand the effect of manual and non-manual work (a characteristic of a job) and vitality at baseline (a personal characteristic), on the change in vitality after retirement or with continued work.

Only a few studies looked at the differential impact of retirement by characteristics of a job. These studies suggest that retirement benefits the health of those who retire from jobs that are stressful, demanding and offer low work-related resources (Eibich 2015; Pinquart and Schindler 2007). Although it is important to study employees’ perceptions about his/her job’s demands and how this affects their health in retirement, for practice implications it is more insightful to differentiate between the types of jobs that are more or less detrimental to health. By this we will be able to identify the types of workers who might experience greater health benefits in retirement. The distinction between manual and non-manual work is deemed to be most relevant. Manual work has been associated with high physical demands and, consequently, physical health impairments (Schaufeli and Taris 2014) and workers in these jobs may benefit more from retiring than non-manual workers. Currently, the statutory pension age in the Netherlands is 66 years and 4 months. This is the mandatory age of retirement. While older Dutch workers prefer to retire early, this option is seldom practised as it is financially disadvantageous (van Solinge and Henkens 2017). Thus, a majority of older workers work up to the statutory pension age. As working contracts are usually terminated once the older workers reach the retirement age, there are also few possibilities of keeping your job after retirement age (Oude Mulders 2019). These measures restrict older workers’ choice in when they can retire. On top of this, similar to the trends in other parts of the Western world, the statutory pension age for *all* workers in the Netherlands is increasing. The statutory pension age, which was 65 years in 1957, will be increased up to 67 years by 2024, based on the increasing trend in life expectancy (van Solinge and Henkens 2017). Even though Dutch retirement-related policies are more generous and well structured compared to other western nations, the increase in statutory pension age could still negatively influence vulnerable groups of older workers. This concern has triggered widespread debate among policy makers on whether statutory pension age should be lower for groups workers in more demanding jobs, such as manual work. In order to contribute to this debate we study whether manual workers benefit more in terms of vitality, energy and fatigue by retiring than non-manual workers? Our hypothesis is that older workers in manual work will experience greater improvements in vitality and energy and greater declines in fatigue after retirement, while older workers in non-manual work may not experience such improvements (*manual work hypothesis*).

Several studies revealed that older workers experiencing poor health might benefit more from retirement in terms of their health (van den Bogaard and Henkens 2018; van den Bogaard et al. 2016). For those older workers, retirement may be a relief from daily work-related burdens and give them more time to focus on health promotion and leisure activities (van den Bogaard et al. 2016). Older workers in manual work may also report low levels of vitality before retirement due to the demanding nature of their jobs. We hypothesize that older workers with poor vitality at baseline may experience greater improvements in vitality after retirement and that this effect partially meditates the hypothesized positive effect of retirement on vitality among manual workers (*baseline vitality hypothesis*).

This study contributes to current literature in three ways. First, it is to the best of our knowledge the first to longitudinally assess the effect of retirement on vitality and its subcomponents. Vitality is a driver of physical and mental well-being and fostering vitality positively impacts organizational productivity and social participation. By analysing vitality and its two subcomponents, energy and fatigue, we add to the current retirement-related literature and we propose practical suggestions on whether interventions should by geared at enhancing energy or reducing fatigue in the workplace. Second, we study how manual work and vitality at baseline influences the effect of retirement on the change in vitality from wave 1 to wave 2. Thereby, we contribute to literature on the heterogeneity of the health consequences of retirement. Furthermore, we contribute to current policy debates on whether public pension age should be increased for all workers, regardless of job type and health status. By providing cues into who would benefit more from retirement, we could demonstrate the importance of flexible public pension and tailored interventions based on job type (van der Mark-Reeuwijk et al. 2019). Third, our 3-year follow-up panel data offer us the unique opportunity to study the heterogeneity in the effects of retirement on vitality based on a sample of older workers aged 60–65 representative for a large part of the Dutch workforce. Approximately half of the sample transitioned into retirement at follow-up.

## Methods

### Population

The NIDI Pension Panel Survey is a Dutch prospective cohort study of employed older workers (and retirees) between the ages of 60 and 65 years (Henkens et al. 2017). This study used data from the NIDI Pension Panel Survey. The survey was administered for all participants over Spring and Summer of 2015 for wave 1 and 2018 for wave 2 in the Netherlands. The NIDI Pension Panel Survey has a stratified random design. In the first step, a sample of organizations was drawn from the files of three large pension funds in the Netherlands (ABP, PfZw and BpBouw). The stratified sample of organizations was drawn along the dimensions of organizational size and sector. The pension funds together represent about 49% of the wage employed workers in the Netherlands, thereby guaranteeing sufficient variation in manual and non-manual labour, job category, educational level and gender (Jaargegevens Individuele Pensioenfondsen 2015). In the second step, older workers aged between 60 and 65 years (birth cohorts 1950–1955), who worked at least 12 h a week, were randomly sampled from the selected organizations. For the first wave, a total of 15,470 questionnaires was sent out to older workers, of which 6793 were completed and returned (net response rate of 44%). Attrition occurred between the first and second waves due to mortality (*N* = 86) and other reasons (e.g. duplication of records, retirement by first wave) (*N* = 12). For the second wave, questionnaires were sent out to 6695 older workers who participated in the first wave. In total 5326 responded (net response rate of 79.6%). Supplementary Table 1 describes and contrasts characteristics of older workers who did and did not respond to the second wave. Older workers who received a shorter version of the questionnaire that did not include all relevant variables (*N* = 513) were excluded from the sample. Missing information on one or more items used to measure vitality led to the exclusion of 326 respondents. A total of 331 respondents indicated that they retired because of health issues. It is likely that the health of these workers worsened between wave 1 and 2 which led them to retire by wave 2. This health decline may have caused declines in vitality after our initial measure of baseline vitality at wave 1. Because we do not have a reliable measure of pre-retirement baseline vitality for this group of older workers, we excluded them from our sample. The final study sample comprised 4156 older workers, all working at wave 1 (wave 1), of whom 1934 (46.5%) retired by wave 2 (wave 2), while 2222 (53.6%) remained in paid employment. Retirement was defined as the complete detachment from workforce, identified by whether the older worker worked any amount of hours for pay. Supplementary Table 2 describes characteristics of older workers who retired and older workers who continued to work by wave 2.

### Measurements

#### Dependent variables

Level of vitality at wave 1 and wave 2 was measured using Medical Outcome Study’s Quality of Life Questionnaire, Short Form-36’s (SF-36) vitality scale. This 4-item measure of vitality questions ‘How much of the time during the past 30 days did you feel: (1) full of energy, (2) tired, (3) worn out and (4) full of pep’ (Ware Jr and Sherbourne 1992) (Supplementary Table 3). Respondents answered all items on a six-point scale, ranging from *constantly* (*1*) to *never* (*6*). Items ‘full of energy’ and ‘full of pep’ were reverse coded. Thereafter, responses for all four items were added to construct a single continuous measure of vitality at wave 2, which was transformed to range from 0 to 100. Higher values indicate higher levels of vitality at wave 2. This measure of vitality demonstrated high reliability at wave 1 (Cronbach’s alpha = 0.81) and at wave 2 (Cronbach’s alpha = 0.82). The minimally important difference (MID) between wave 1 and wave 2 for vitality measured using the SF-36 vitality scale for groups of participants was recommended to be held at 5 points on a 0–100 scale (MID) (Bjorner et al. 2007). This measure will be used to ascertain whether the change in vitality levels is of clinical relevance.

In addition to measuring vitality as a single construct, we also separated it to study the positive and negative states within vitality: energy and fatigue, as has been suggested in the literature (Deng et al. 2015; Ware Jr and Sherbourne 1992). Energy at wave 2 was measured using the two items ‘full of energy’ and ‘full of pep’. A single continuous measure of energy ranging from 0 to 100 was constructed. Higher values indicated higher levels of energy at wave 2. After reverse coding, the two items ‘tired’ and ‘worn out’, they were used construct a single continuous measure of fatigue at wave 2 which ranged from 0 to 100. Higher values reflected higher levels of fatigue at wave 2. These 2-item scales of energy (wave 1: Cronbach’s alpha = 0.74, wave 2: Cronbach’s alpha = 0.76) and fatigue (wave 1: Cronbach’s alpha = 0.82, wave 2: Cronbach’s alpha = 0.81) demonstrated high reliability.

#### Independent variables

All respondents were employed at wave 1. Older workers’ transition into retirement or their continuance of work between wave 1 and wave 2 was assessed by inquiring ‘Which situation applies to you?’ at wave 2. Responses were expressed by choosing between *I work for pay* and *I am fully retired*. Based on the responses, we created a dichotomized variable of retirement status at wave 2. We considered anyone who was employed in their career job, bridge employment, part-time work or short-term work as *working* (*0*) and anyone who was fully retired and engaged in no paid work as *retired* (*1*).

Manual work was measured at wave 1 using the item ‘In which category could your job or profession be grouped?’. Respondents chose one among nine categories of the International Standard Classification of Occupation (Ganzeboom 2010). The categories ranged from *higher intellectual or free profession* (*1*) to *agricultural profession* (*9*). Based on these responses, we created a dichotomized variable of manual work, which we coded 1 if respondents’ jobs consisted of manual work based on the International Standard Classification of Occupation (Ganzeboom 2010).

#### Covariates

We controlled for several demographic, health-related and work-related factors, all measured at wave 1. Age, in years, was treated as continuous variable. Sex (*1* = *male*) and presence of a partner (*1* = *partner present*) were represented by dichotomized variables. Educational attainment was measured in seven categories: *primary school* (*1*)*, lower vocational education* (*2*)*, lower general secondary education* (*3*)*, intermediate vocational education* (*4*)*, upper general secondary education* (*5*)*, higher vocational education* (*6*) and *university graduate* (*7*). Subsequently, we grouped categories together to create three dichotomized variables: low (1,2,3), moderate (4,5) and high (6,7) educational attainment. Similarly, wealth was measured in seven categories: < *5000 euros* (*1*) to > *500,000 euros* (*7*). Thereafter, it was grouped into three dichotomized variables: low (less than 50,000 euros), moderate (between 50,000 and 250,000 euros) and high (more than 500,000 euros) levels of wealth. Caregiving responsibilities were variable and were coded 1 if respondents replied affirmatively to the question ‘Do you provide help to family members or friends who are ill or in need of help?’.

Additionally, we adjusted for whether respondents suffered from chronic health condition/s (CHCs). CHC was dichotomized and coded 1 if respondents experienced one or more CHC. Work-related factors included: full-time employment, supervisory position, size of organization and sector. Full-time employment and supervisory position were dichotomized. Respondents who worked for or over 36 h a week were coded 1 on full-time employment. Supervisory position was coded 1 if respondents said *yes* to the question ‘Do you have a supervisory position?’. Organizational size and sector were categorical variables with three categories each. Organizations were separated by size into small (< 50 employees), medium (50–250 employees) and large (> 250 employees). Organizations belonged to three sectors: government and education, construction and health and welfare. Descriptive statistics and coding and psychometric properties of all variables, before standardization, are presented in Supplementary Table 4.

### Analyses

To examine the effect of retirement and manual work on the change in vitality, energy and fatigue between wave 1 and wave 2, we conducted conditional change ordinal least square (OLS) regression analyses. In conditional change models, the dependent variable measured at wave 2 is regressed on levels of the dependent variable measured at wave 1, independent variables and control variables (Aickin 2009). Including wave 1 values of the dependent variable in the regression analysis controls for possible ceiling effects. In our conditional change models, the scores of vitality, energy and fatigue at the second wave were the dependent variables. We regressed these dependent variables against their wave 1 s values, retirement status at wave 2 and manual work. The resulting effects could be interpreted as change effects from wave 1 to wave 2.

Model 1, Model 2 and Model 3 examined the effects of retirement, manual work and wave 1 vitality, energy or fatigue on the change in vitality (model 1), energy (model 2) or fatigue (model 3) between wave 1 and wave 2. Model 1a and model 1b predict the change in vitality after retirement. The key difference between models 1a and 1b is that model 1b includes the interaction term between wave 1 vitality and retirement status on the change in vitality after retirement, while model 1a does not.

All dependent variables were standardized before regression analyses. This allowed the interpretation of dichotomized variables (specifically retirement status and manual work) as Cohen’s *d* effect sizes (Cohen 2013). Missing data of all variables, except vitality, fatigue and energy, were imputed using single stochastic regression imputation (Enders 2010). As item non-response was under 5% for any single item in our data, our use of a less vigorous missing data imputation method was acceptable (Little et al. 2014).

## Results

Table 1 presents the results of the conditional change OLS regression analyses. It examined the effects of retirement, manual work and wave 1 vitality, energy or fatigue on the change in vitality (model 1), energy (model 2) and fatigue (model 3) from wave 1 and wave 2.

**Table 1:** Effects of retirement status at wave 2 and manual work on change in vitality, energy or fatigue between wave 1 and wave 2 (*N* = 4156)

Variables	Model 1: vitality at w2				Model 2: energy at w2		Model 3: fatigue at w2	
	1a: without vitality at w1 × retirement status at w2		1b: with vitality at w1 × retirement status at w2					
	Coef.	SE	Coef.	SE	Coef.	SE	Coef.	SE
Retirement status at w2 (1 = retired)	0.29**	0.03	0.29**	0.03	0.16**	0.03	− 0.33**	0.03
Vitality at w1	0.55**	0.01	0.61**	0.02				
Vitality at w1 × retirement status at w2			− 0.12**	0.02				
Energy at w1					0.49**	0.02		
Energy at w1 × retirement status at w2					− 0.02	0.03		
Fatigue at w1							0.60**	0.02
Fatigue at w1 × retirement status at w2							− 0.16**	0.02
Manual work (1 = manual work)	− 0.21**	0.05	− 0.21**	0.05	− 0.17*	0.05	0.20**	0.05
Manual work × retirement status at w2	0.14*	0.06	0.13*	0.06	0.08	0.07	− 0.16*	0.06
*Demographic controls* (*w1*)								
Age	0.03**	0.01	0.03**	0.01	0.03*	0.01	− 0.04**	0.01
Sex (1 = male)	− 0.00	0.03	− 0.00	0.03	− 0.03	0.03	− 0.03	0.03
Presence of partner (1 = partner present)	0.05	0.03	0.05ª	0.03	0.10*	0.03	− 0.00	0.03
Educational attainment (*ref* = *low*)								
Moderate	0.02	0.04	0.01	0.04	0.07ª	0.04	0.03	0.04
High	0.08*	0.04	0.08*	0.04	0.13*	0.04	− 0.03	0.04
Wealth (ref = low)								
Moderate	0.07*	0.03	0.07*	0.03	0.04	0.03	− 0.09*	0.03
High	0.11*	0.03	0.11*	0.03	0.11*	0.04	− 0.10*	0.04
Caregiving responsibilities (1 = provides care)	− 0.00	0.02	− 0.01	0.02	0.00	0.03	0.01	0.02
*Health*-*related controls* (*w1*)								
Having a chronic health condition/s (1 = yes)	− 0.13**	0.03	− 0.13**	0.03	− 0.14**	0.03	0.14**	0.03
*Work*-*related controls* (*w1*)								
Full-time employment (1 = employed full-time)	0.04	0.03	0.04	0.03	0.05ª	0.03	− 0.02	0.03
Supervisory position (1 = supervisory position)	− 0.06*	0.03	− 0.06*	0.03	− 0.06ª	0.03	0.04	0.03
Organizational size (ref = small)								
Medium	0.00	0.03	− 0.00	0.03	− 0.03	0.04	− 0.03	0.04
Large	− 0.00	0.04	− 0.00	0.04	− 0.03	0.04	− 0.02	0.04
Organizational sector (ref = government and education)								
Construction sector	0.03	0.04	0.03	0.04	0.04	0.04	0.00	0.04
Health and welfare sectors	0.01	0.03	0.01	0.03	0.03	0.03	0.00	0.03
Constant	− 2.12**	0.55	− 2.24**	0.55	− 2.15**	0.59	2.43**	0.56
Adjusted *R*^2^	0.38		0.39		0.29		0.36	

**p < 0.001, *p < 0.05, ª*p* < 0.10, *Coef.*coefficient, *SE* standard error, *w1* wave 1, *w2* wave 2

### Vitality

**Fig. 1:**
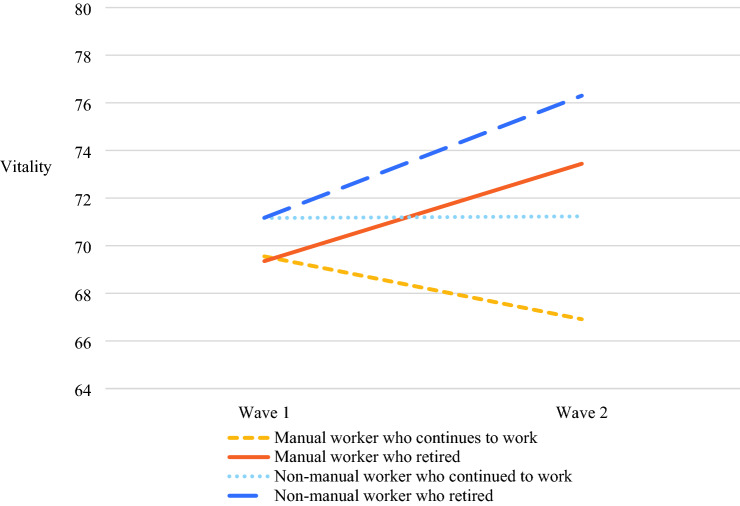
Effects of retirement and manual work on vitality from wave 1 to wave 2 among older workers and retirees

Model 1a showed that vitality increased after transitioning into retirement (Cohen’s *d* = 0.34, *p* < 0.001). Model 1a also revealed that being engaged in manual work is associated with a decrease in vitality among all older workers (Cohen’s *d* = − 0.22, *p* < 0.001). Manual workers who retired experienced a greater increase in vitality than non-manual workers who retired. This is evident from the significant positive interaction between manual work and retirement on the change in vitality between wave 1 and wave 2 in model 1a (Cohen’s *d* = 0.12, *p* < 0.05). Figure 1 illustrates the differences in the change in vitality between manual and non-manual workers. Manual workers have lower levels of vitality at baseline (wave 1) and showed an increase in vitality between wave 1 and wave 2 for those who retired and a decrease in vitality for those who remained employed. Also, non-manual workers who retired showed improvements in vitality compared to those who remained employed, but to a lesser extent. For both retired and non-retired workers the MID for vitality exceeded the 5 points, for both manual workers (6.53) and non-manual workers (5.07) affirming these differences in vitality to be clinically relevant. The *manual work hypothesis* was confirmed.

**Fig. 2:**
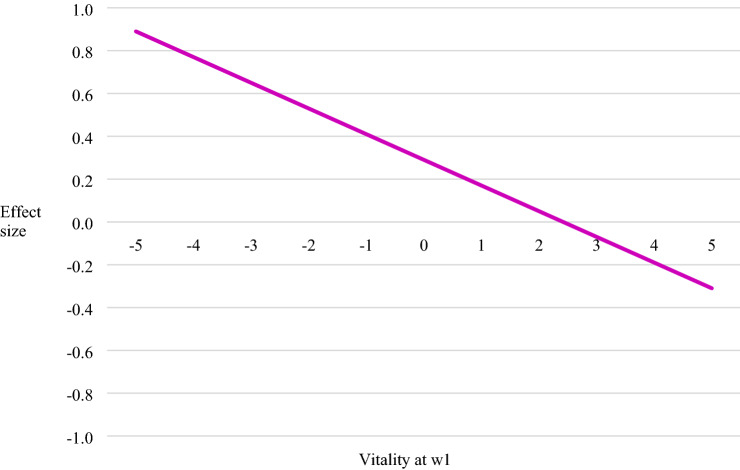
Effect of retirement on older workers’ vitality at wave 2 by vitality at wave 1

In Model 1b we extended the baseline model (1a) by including the interaction term between wave 1 vitality and retirement status. This model showed that the effect of retirement on changes in vitality between wave 1 and wave 2 is highly dependent on the baseline level of vitality at wave 1. Older workers with low levels of vitality at wave 1 benefit much more from retirement than workers with high levels of vitality at wave 1 (*b* = − 0.15, *p* < 0.001). This is illustrated in Fig. 2, which shows that the size of the effect of retirement on the change in vitality was much stronger for older workers experiencing low levels of vitality at wave 1 (*b* = 0.95, *p* < 0.001) than for those experiencing high levels of vitality at wave 1.

Furthermore, the addition of this interaction effect in model 1b leads to the reduction in the size of the interaction effect between manual work and retirement (from Cohen's *d* = 0.12, *p* < 0.05 in model 1a to *b* = 0.10, *p* < 0.10 in model 1b). This provides evidence that the improvement of vitality upon retirement among manual workers might be traced back to the fact that manual workers have lower baseline levels of vitality. This confirms the *baseline vitality hypothesis.*

### Energy

Model 2 showed that retirement is associated with increased energy levels (Cohen’s *d* = 0.20, *p* < 0.001). There was no significant difference in the change in energy levels from wave 1 to wave 2 between manual and non-manual workers; the interaction term between manual work and retirement status was not significant (Cohen’s *d* = − 0.02, *p* > 0.05). Moreover, the interaction term between retirement status and wave 1 energy level on the change in energy levels from wave 1 to wave 2 was not significant.

### Fatigue

**Fig. 3:**
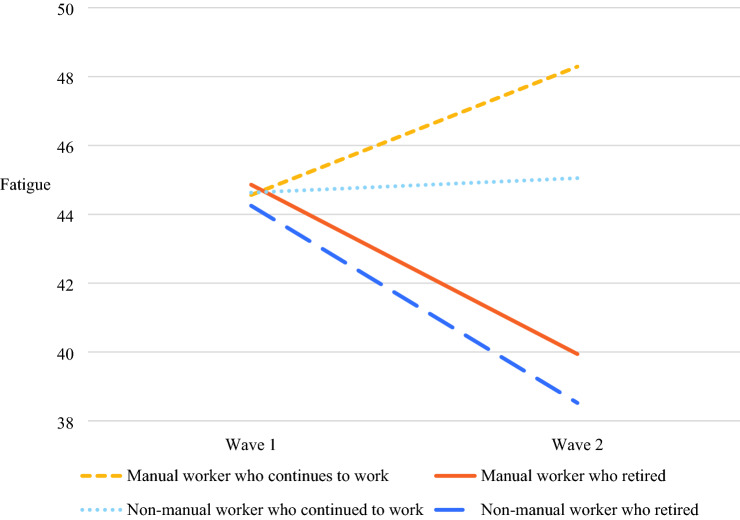
Effects of retirement and manual work on the change in fatigue from wave 1 to wave 2 among older workers and retirees

Model 3 reveals that retirement is associated with reduced fatigue levels (Cohen’s *d* = − 0.40, *p* < 0.001). Manual workers who retired experienced a greater decrease in fatigue than non-manual workers who retired (*b* = − 0.14, *p* < 0.05). This confirms the *manual work hypothesis*. Figure 3 illustrates this interaction effect. Older manual workers who remained employed experienced increases in fatigue between wave 1 and wave 2, whereas their retired counterparts reported decreases in fatigue between both waves. Older non-manual workers who remained employed reported a slight increase in fatigue at wave 2, whereas those who retired experienced decreases in fatigue at wave 2.

Moreover, our findings showed that the negative effect of retirement on fatigue is much stronger for older workers who report high levels of fatigue at baseline. This is affirmed by the significant negative interaction between retirement status and wave 1 fatigue on the change in fatigue from wave 1 to wave 2 (*b* = − 0.20, *p* < 0.001). This confirms the *baseline fatigue hypothesis.*

## Discussion

Vitality is an important aspect of health. This study is, to the best of our knowledge, one of the first to describe the effect of retirement on vitality and its subcomponents, energy and fatigue. We demonstrate how this effect varies based on wave 1 job type and wave 1 vitality. Our findings reveal that retirement is associated with a clinically relevant increase in vitality. This increase was greatest for older workers in manual work and older workers experiencing low levels of vitality at wave 1. Additionally, we demonstrate that retirement reduces fatigue, more so for older workers who retired from manual work and those who were fatigued before retirement. No such effects were found for energy.

By disentangling the subcomponents of vitality, we demonstrate that fatigue, not energy, is the driver for change in vitality from wave 1 to wave 2. This could be due to the association between fatigue and burnout. Many studies have shown that fatigue and burnout share similar symptoms and consequences (Leone et al. 2011), and that fatigue acts as a predictor of burnout (Raftopoulos et al. 2012). Such an association has not been found between energy and burnout. Our findings offer insights into how vitality can be improved in practice: either by promoting a general notion of vitality or, better yet, by targeting the reduction of fatigue. Worksite vitality interventions, such as the Vital@Work intervention, worksite yoga and exercise interventions (de Vries et al. 2017; Strijk et al. 2012) and mobilization interventions (Mailey et al. 2017), have been found to improve vitality among older workers. In regards of fatigue at the workplace, interventions that target the improvement of sleep have been shown to successfully reduce fatigue among workers (Sadeghniiat-Haghighi and Yazdi 2015). Additionally, workplaces that promote psychosocial safety climates have been associated with better recovery among workers when fatigued (Garrick et al. 2014). Building on these findings, organizations may offer their older workforce with effective (worksite) vitality and fatigue interventions that could sustain and promote the health of older workers, while they are at work.

The effect of retirement on vitality is not uniform across all older workers. Compared to non-manual workers, manual workers who retired experienced greater health benefits (in terms of vitality and fatigue), while manual workers who continued to work experienced greater health declines. Manual work has been associated with a greater physical workload, greater physical job demands and less control over their work (Raittila et al. 2017; Schreuder et al. 2008). Manual workers are also engaged in repetitive, risky and strenuous movements on a daily basis (Melchior et al. 2006). These unfavourable working conditions have been shown to fuel stress and unhealthy behaviours, leading to health inequalities between manual and non-manual workers (Peretti-Watel et al. 2009). Moreover, manual workers were found to have lower work ability than non-manual workers, which in turn was associated with long-term sickness absence from work (Schouten et al. 2015). In addition, it is a challenge to develop worksite vitality interventions that target manual workers. Most interventions that exist today focus on and cater to non-manual workers (de Vries et al. 2017; Mailey et al. 2017; Strijk et al. 2012, 2013). Our results are interesting in view of the ongoing debate about increasing retirement ages in ageing countries. This increase may be more challenging for some groups of older workers, such as manual workers, compared to others. A study that questioned employer’s perspectives on the increasing retirement age in The Netherlands revealed that employers from construction and industry sectors were highly concerned about the physical capabilities of their older employees to work longer and that they overwhelmingly supported the lowering of the public pension age for manual workers (van Dalen et al. 2019). Instead of sticking to a one size fits all approach, policy makers may consider introducing job type-based and health-based flexible pension options that accommodate the heterogeneities in the health consequences of retirement (Health and Working Longer 2018; van Dalen et al. 2019). Moreover, organizations may consider reducing the extent of job demand and job burdens on older manual workers by altering their job roles. Likewise, future research could contribute by developing worksite vitality or fatigue reduction interventions that are tailored to the unique difficulties and needs faced by manual workers.

The health effects of retirement also depended on how healthy older workers are before retirement: older workers experiencing poor vitality before retirement experienced greater surges in vitality after retirement. Poor health among older workers is a by-product of ageing (McMahan and Sturz 2006). A significant proportion of older workers suffer from health-related work limitations (Vanajan et al. 2019). Past studies have described ways in which organizations can accommodate older workers with poor health and health-related work limitations. For example, organizations with psychologically safe workplace climates are associated with fewer health-related work limitations (Vanajan et al. 2019). Similarly, the provision of flexible work arrangements is shown to help reduce the negative impact of poor health among older workers (Moen et al. 2016; Vanajan et al. 2019). Moreover, our result calls to view occupational health and safety through the life course perspective (Amick et al. 2016). Occupational health and safety professionals should consider how earlier (working) life influences later life health outcomes (Amick et al. 2016). In this case, how vitality earlier in life (together with numerous other labour market, work and health trajectories) could influence vitality at work and after retirement in later life.

The core strength of this study is that it goes beyond existing literature in the health-retirement nexus by simultaneously studying the effect of retirement on vitality and its subcomponents and by examining how this effect may vary based on manual versus non-manual work and baseline vitality of older workers. This is done using an innovative new cohort study with two waves, the second of which was a 3-year follow-up. This cohort study includes data on many older workers who made the transition into retirement and who are still working. In addition to this, the findings provide insights into policy and practice implications on an organizational and governmental level.

This study is not without its limitations. This study is conducted in the Netherlands, where retirement benefits are generous and well structured. Our results, therefore, may not be generalizable to nations with dissimilar pension structures. It is also interesting to further elucidate the mechanisms through which retirement increases vitality. The mechanisms might not only relate to the lack of work-related burdens, but also to more rest, more leisure time or to the development of positive health behaviour.

The extension of working lives is a key policy and public health priority in the western world. Linking public pension age to an averaged measure of life expectancy is more likely to increase the burden on already disadvantaged groups of older workers (Krekula and Vickerstaff 2020). Our findings show that older workers in manual work and those experiencing low vitality and high fatigue may suffer from an extension of working lives. Current work-related and retirement-related policies focus on the average worker, without making any distinctions between workers in manual jobs and workers who no not face any physical strain in their work. This study might stimulate policy makers to consider the differences in the ways in which specific groups of older workers react to an increased retirement age, in order to develop inclusive and sensitive retirement-related policies (Krekula and Vickerstaff 2020). In any case, it is beneficial to provide older workers with effective interventions aimed at improving vitality and reducing fatigue. Additionally, it is advantageous to accommodate age-related detriments in health and work ability through flexible work arrangements and supportive organizational climates. The timely provision of these interventions, may not only improve the vitality of older workers, but also the vitality of organizations and the society at large.

## Electronic supplementary material

Below is the link to the electronic supplementary material.

Supplementary material 1 (DOCX 34 kb)

## Data Availability

The data that support the findings of this study are available from the corresponding author, A.V., upon reasonable request.

## References

[CR1] Aickin M (2009). Dealing with change: using the conditional change model for clinical research. Perm J.

[CR2] Aleksandra Basinska B, Wiciak I, Maria Dåderman A (2014). Fatigue and burnout in police officers: the mediating role of emotions. Polic Int J Police Strateg Manag.

[CR3] Amick BC, McLeod CB, Bultmann U (2016). Labor markets and health: an integrated life course perspective. Scand J Work Environ Health.

[CR4] Bjorner JB, Wallenstein GV, Martin MC, Lin P, Blaisdell-Gross B, Tak Piech C, Mody SH (2007). Interpreting score differences in the SF-36 Vitality scale: using clinical conditions and functional outcomes to define the minimally important difference. Curr Med Res Opin.

[CR5] Butterworth P, Gill SC, Rodgers B, Anstey KJ, Villamil E, Melzer D (2006). Retirement and mental health: analysis of the Australian national survey of mental health and well-being. Soc Sci Med.

[CR6] Carmeli A, Härtel CE, Zerbe WJ, Ashkanasy NM (2009). Positive work relationships, vitality, and job performance. Emotions in groups, organizations and cultures.

[CR7] Cohen J (2013). Statistical power analysis for the behavioral sciences.

[CR8] de Vries JD, van Hooff ML, Guerts SA, Kompier MA (2017). Exercise to reduce work-related fatigue among employees: a randomized controlled trial. Scand J Work Environ Health.

[CR9] Deng N, Guyer R, Ware JE (2015). Energy, fatigue, or both? A bifactor modeling approach to the conceptualization and measurement of vitality. Qual Life Res.

[CR10] Eibich P (2015). Understanding the effect of retirement on health: mechanisms and heterogeneity. J Health Econ.

[CR11] Elçi M, Yildiz B, Erdilek Karabay M (2018). How burnout affects turnover intention? The conditional effects of subjective vitality and supervisor support. Int J Organ Leadersh.

[CR12] Enders CK (2010). Applied missing data analysis.

[CR13] Ganzeboom HBG (2010) A new International Socio-Economic Index (ISEI) of occupational status for the International Standard Classification of Occupation 2008 (ISCO-08) constructed with data from the ISSP 2002–2007. In: Paper presented at the annual conference of international social survey programme, Lisbon

[CR14] Garrick A, Mak AS, Cathcart S, Winwood PC, Bakker AB, Lushington K (2014). Psychosocial safety climate moderating the effects of daily job demands and recovery on fatigue and work engagement. J Occup Organ Psychol.

[CR15] Health and Working Longer (2018) Health Council of the Netherlands, The Hague

[CR16] Henkens K, Van Solinge H, Damman M, Dingemans E (2017). Design and codebook of the NIDI Pension Panel Study (NPPS) first wave, 2015.

[CR17] Hennekam S (2016). Vitality of older workers and its relationship with performance, career satisfaction and career success. Manag Avenir.

[CR18] Henning G, Lindwall M, Johansson B (2016). Continuity in well-being in the transition to retirement. GeroPsych.

[CR19] Jaargegevens Individuele Pensioenfondsen (2015) De Nederlandsche Bank: https://www.dnb.nl/statistiek/statistieken-dnb/financiele-instellingen/pensioenfondsen/gegevens-individuele-pensioenfondsen/index.jsp. Accessed 22 Feb 2017

[CR20] Kark R, Carmeli A (2009). Alive and creating: the mediating role of vitality and aliveness in the relationship between psychological safety and creative work involvement. J Organ Behav Int J Ind Occup Organ Psychol Behav.

[CR21] Krekula C, Vickerstaff S (2020). The ‘older worker’ and the ‘ideal worker’: a critical examination of concepts and categorisations in the rhetoric of extending working lives.

[CR22] Leone SS, Wessely S, Huibers MJH, Knottnerus JA, Kant IJ (2011). Two sides of the same coin? On the history and phenomenology of chronic fatigue and burnout. Psychol Health.

[CR23] Little TD, Jorgensen TD, Lang KM, Moore EW (2014). On the joys of missing data. J Pediatr Psychol.

[CR24] Mailey EL, Rosenkranz SK, Ablah E, Swank A, Casey K (2017). Effects of an intervention to reduce sitting at work on arousal, fatigue, and mood among sedentary female employees. J Occup Environ Med.

[CR25] McMahan S, Sturz D (2006). Implications for an aging workforce. J Educ Bus.

[CR26] Melchior M, Roquelaure Y, Evanoff B, Chastang JF, Ha C, Imbernon E, Leclerc A (2006). Why are manual workers at high risk of upper limb disorders? The role of physical work factors in a random sample of workers in France (the Pays de la Loire study). Occup Environ Med.

[CR27] Moen P, Kojola E, Schaefers K (2016). Organizational change around an older workforce. Gerontol.

[CR28] Oude Mulders J (2019). Attitudes about working beyond normal retirement age: the role of mandatory retirement. J Aging Soc Policy.

[CR29] Peretti-Watel P, Constance J, Seror V, Beck F (2009). Working conditions, job dissatisfaction and smoking behaviours among French clerks and manual workers. J Occup Environ Med.

[CR30] Pinquart M, Schindler I (2007). Changes of life satisfaction in the transition to retirement: a latent-class approach. Psychol Aging.

[CR31] Raftopoulos V, Charalambous A, Talias M (2012). The factors associated with the burnout syndrome and fatigue in Cypriot nurses: a census report. BMC Public Health.

[CR32] Raittila S, Rahkonen O, Lahelma E, Alho J, Kouvonen A (2017). Occupational class differences in trajectories of working conditions in women. Int J Environ Res Public Health.

[CR33] Sadeghniiat-Haghighi K, Yazdi Z (2015). Fatigue management in the workplace. Ind Psychiatry J.

[CR34] Schaufeli WB, Taris TW, Bauer GF, Hämmig O (2014). A critical review of the job demands-resources model: implications for improving work and health. Bridging occupational, organizational and public health.

[CR35] Schouten LS, Joling CI, van der Gulden JWJ, Heymans MW, Bültmann U, Roelen CAM (2015). Screening manual and office workers for risk of long-term sickness absence: cut-off points for the Work Ability Index. Scand J Work Environ Health.

[CR36] Schreuder KJ, Roelen CAM, Koopmans PC, Groothoff JW (2008). Job demands and health complaints in white and blue collar workers. Work.

[CR37] Strijk JE, Proper KI, van der Beek AJ, van Mechelen W (2012). A worksite vitality intervention to improve older workers’ lifestyle and vitality-related outcomes: results of a randomised controlled trial. J Epidemiol Community Health.

[CR38] Strijk JE, Proper KI, van Mechelen W, van der Beek AJ (2013). Effectiveness of a worksite lifestyle intervention on vitality, work engagement, productivity, and sick leave: results of a randomized controlled trial. Scand J Work Environ Health.

[CR39] van Dalen HP, Henkens K, Oude Mulders J (2019). Increasing the public pension age: employers’ concerns and policy preferences. Work Aging Retire.

[CR40] van den Bogaard L, Henkens K (2018). When is quitting an escape? How different job demands affect physical and mental health outcomes of retirement. Eur J Public Health.

[CR41] van den Bogaard L, Henkens K, Kalmijn M (2016). Retirement as a relief? The role of physical job demands and psychological job stress for effects of retirement on self-rated health. Eur Sociol Rev.

[CR42] van der Heide I, van Rijn RM, Robroek SJ, Burdorf A, Proper KI (2013). Is retirement good for your health? A systematic review of longitudinal studies. BMC Public Health.

[CR43] van der Mark-Reeuwijk KG, Weggemans RM, Bultmann U, Burdorf A, Deeg DJ, Geuskens G, Lindeboom M (2019). Health and prolonging working lives: an advisory report of the Health Council of The Netherlands. Scand J Work Environ Health.

[CR44] van Scheppingen AR, de Vroome EM, ten Have KC, Zwetsloot GI, Bos EH, van Mechelen W (2014). Motivations for health and their associations with lifestyle, work style, health, vitality, and employee productivity. J Occup Environ Med.

[CR45] Van Solinge H (2007). Health change in retirement: a longitudinal study among older workers in the Netherlands. Res Aging.

[CR46] van Solinge H, Henkens K (2017). Older workers’ emotional reactions to rising retirement age: the case of the Netherlands. Work Aging Retire.

[CR47] van Steenbergen E, van Dongen JM, Wendel-Vos GCW, Hildebrandt VH, Strijk JE (2016). Insights into the concept of vitality: associations with participation and societal costs. Eur J Public Health.

[CR48] Vanajan A, Bültmann U, Henkens K (2019). Health-related work limitations among older workers—the role of flexible work arrangements and organizational climate. Gerontol.

[CR49] Vanajan A, Bültmann U, Henkens K (2020). Why do older workers with chronic health conditions prefer to retire early?. Age Ageing.

[CR50] Ware J, Sherbourne CD (1992). The MOS 36-item short-form health survey (SF-36): I. Conceptual framework and item selection. Med Care.

