# Supplementary material for: Do older manual workers benefit in vitality after retirement? Findings from a 3-year follow-up panel study
Source: Eur J Ageing. 2020 Nov 4;18(3):369–79. doi: 10.1007/s10433-020-00590-7 (PMC8377110; doi:10.1007/s10433-020-00590-7)
Supplement: Supplementary file 1 — Supplementary material 1 (DOCX 34 kb) [file 10433_2020_590_MOESM1_ESM.docx]

Supplementary file

Table 1.

| Variables | Drop outs from w1 to w2  (N = 1,308) | | Respondents at wave 2  (N = 4,972) | |
| --- | --- | --- | --- | --- |
|  | Mean | SD | Mean | SD |
|  |  |  |  |  |
| Vitality w1 | 68.08 | 15.09 | 69.61 | 14.05 |
| Energy w1 | 67.06 | 17.23 | 68.66 | 16.07 |
| Fatigue w1 | 47.40 | 16.70 | 45.89 | 15.33 |
|  |  |  |  |  |
| Manual work (dichotomized, 0-1) | 0.25 | 043 | 0.19 | 0.39 |
|  |  |  |  |  |
| Demographic controls |  |  |  |  |
| Age (scale, 0-100) | 61.97 | 1.62 | 62.03 | 1.59 |
| Male sex (dichotomized, 0-1) | 0.57 | 0.50 | 0.55 | 0.50 |
| Partner present (dichotomized, 0-1) | 0.88 | 0.33 | 0.81 | 0.39 |
| Educational attainment (categorical, 1-3) |  |  |  |  |
| Low (1) | 0.32 | 0.47 | 0.26 | 0.44 |
| Moderate (2) | 0.28 | 0.45 | 0.26 | 0.44 |
| High (3) | 0.40 | 0.49 | 0.48 | 0.50 |
| Wealth (categorical, 1-3) |  |  |  |  |
| Low (1) | 0.34 | 0.47 | 0.32 | 0.47 |
| Moderate (2) | 0.46 | 0.50 | 0.47 | 0.50 |
| High (3) | 0.20 | 0.40 | 0.21 | 0.41 |
| Caregiving responsibilities (dichotomized, 0-1) | 0.61 | 0.49 | 0.61 | 0.49 |
|  |  |  |  |  |
| Health-related controls |  |  |  |  |
| Having a chronic health condition (dichotomized, 0-1) | 0.70 | 0.46 | 0.68 | 0.46 |
|  |  |  |  |  |
| Work-related controls |  |  |  |  |
| Full-time employment (dichotomized, 0-1) | 0.49 | 0.50 | 0.47 | 0.50 |
| Supervisory position (dichotomized, 0-1) | 0.25 | 0.43 | 0.25 | 0.43 |
| Organizational size (categorical, 1-3) |  |  |  |  |
| Small (1) | 0.16 | 0.37 | 0.16 | 0.37 |
| Medium (2) | 0.45 | 0.50 | 0.44 | 0.50 |
| Large (3) | 0.39 | 0.49 | 0.40 | 0.49 |
| Organizational sector (categorical, 1-3) |  |  |  |  |
| Government and education sectors (1) | 0.41 | 0.49 | 0.49 | 0.50 |
| Construction (2) | 0.24 | 0.43 | 0.20 | 0.40 |
| Health and welfare (3) | 0.34 | 0.48 | 0.31 | 0.46 |

Characteristics of respondents who dropped out between waves 1 and 2 (N = 1,308) and those who responded to wave 2 (N = 4,972)

Table 2.

Characteristics of older workers working at wave 2 (N = 2,222) vs. older workers who retired by wave 2 (N = 1,934)

| Variable | Working at wave 2 | | Retired at wave 2 | |
| --- | --- | --- | --- | --- |
|  | Mean | *SD* | Mean | *SD* |
|  |  |  |  |  |
| Vitality w1 | 70.87 | 13.06 | 70.85 | 13.14 |
| Energy w1 | 69.70 | 15.44 | 69.39 | 15.61 |
| Fatigue w1 | 44.62 | 14.29 | 44.36 | 14.43 |
|  |  |  |  |  |
| Manual work (dichotomized, 0-1) | 0.18 | 0.38 | 0.18 | 0.38 |
|  |  |  |  |  |
| *Demographic controls at w1* |  |  |  |  |
| Age (scale, 0-100) | 61.21 | 1.18 | 62.99 | 1.48 |
| Male sex (dichotomized, 0-1) | 0.54 | 0.50 | 0.56 | 0.50 |
| Partner present (dichotomized, 0-1) | 0.80 | 0.40 | 0.82 | 0.39 |
| Educational attainment (categorical, 1-3) |  |  |  |  |
| Low (1) | 0.24 | 0.43 | 0.25 | 0.43 |
| Moderate (2) | 0.26 | 0.44 | 0.25 | 0.44 |
| High (3) | 0.50 | 0.50 | 0.50 | 0.50 |
| Wealth (categorical, 1-3) |  |  |  |  |
| Low (1) | 0.35 | 0.48 | 0.27 | 0.44 |
| Moderate (2) | 0.46 | 0.50 | 0.46 | 0.50 |
| High (3) | 0.18 | 0.39 | 0.27 | 0.45 |
| Caregiving responsibilities (dichotomized, 0-1) | 0.61 | 0.49 | 0.61 | 0.49 |
|  |  |  |  |  |
| *Health-related controls at w1* |  |  |  |  |
| Having a chronic health condition (dichotomized, 0-1) | 0.65 | 0.48 | 0.69 | 0.46 |
|  |  |  |  |  |
| *Work-related controls at w1* |  |  |  |  |
| Full-time employment (dichotomized, 0-1) | 0.50 | 0.50 | 0.45 | 0.50 |
| Supervisory position (dichotomized, 0-1) | 0.25 | 0.43 | 0.26 | 0.44 |
| Organizational size (categorical, 1-3) |  |  |  |  |
| Small (1) | 0.16 | 0.37 | 0.16 | 0.37 |
| Medium (2) | 0.43 | 0.50 | 0.46 | 0.50 |
| Large (3) | 0.41 | 0.49 | 0.38 | 0.49 |
| Organizational sector (categorical, 1-3) |  |  |  |  |
| Government and education sectors (1) | 0.48 | 0.50 | 0.51 | 0.50 |
| Construction (2) | 0.19 | 0.39 | 0.21 | 0.41 |
| Health and welfare (3) | 0.33 | 0.47 | 0.28 | 0.45 |

Table 3.

Items of the Vitality scale

| How much of the time in the last 30 days did you feel: | N | Mean | *SD* |
| --- | --- | --- | --- |
| 1. Full of energy | 4,156 | 4.20 | 1.00 |
| 1. Tired | 4,156 | 3.96 | 0.87 |
| 1. Worn out | 4,156 | 4.70 | 1.01 |
| 1. Full of pep | 4,156 | 4.14 | 1.11 |

Table 4.

Descriptive statistics of (unstandardized) variables in a sample of 4,156 older workers and retirees (N = 4,156)

| Variables | Mean | *SD* | Coding and Psychometric properties | Wording of survey question |
| --- | --- | --- | --- | --- |
|  |  |  |  |  |
| Vitality at w1 | 70.86 | 13.09 | Scale variables, ranging from 0 to 100 | How much of the time during the past 30 days did you feel:   1. full of energy 2. tired 3. worn out 4. full of pep   (6 answer categories on a Likert scale ranging from 1=constantly to 6=never) |
| Vitality at w2 | 72.94 | 13.19 |  |  |
| Energy at w1 | 69.56 | 15.52 | Scale variables, ranging from 0 to 100 | How much of the time during the past 30 days did you feel:   1. full of energy 2. full of pep   (6 answer categories on a Likert scale ranging from 1=constantly to 6=never) |
| Energy at w2 | 71.66 | 15.49 |  |  |
| Fatigue at w1 | 44.50 | 14.35 | Scale variables, ranging from 0 to 100 | How much of the time during the past 30 days did you feel:   1. tired 2. worn out   (6 answer categories on a Likert scale ranging from 1=constantly to 6=never) |
| Fatigue at w2 | 42.44 | 14.37 |  |  |
| Retirement status at w2 | 0.49 | 0.50 | Dichotomized variable:  1=Retired  0=Still working | Which situation applies to you?  (2 answer categories: 1=I work for pay, 2=I do not work for pay any longer) |
| Manual work | 0.18 | 0.38 | Dichotomized variable:  1=Manually work  0=Non-manual work | Based on the International Standard Classification of Occupation, in which category could your job be grouped? |
|  |  |  |  |  |
| *Demographic controls* |  |  |  |  |
| Age | 62.03 | 1.60 | Scale variable: ranging from 60 to 65 years | In what year were you born? (age in years were calculated) |
| Sex | 0.55 | 0.50 | Dichotomized variable:  1=Male  0=Female | Are you a man or woman?  (2 answer categories: 1=man, 2=women) |
| Presence of partner | 0.81 | 0.39 | Dichotomized variable:  1=Partner present  0=No partner present | Do you have a partner?  (4 answer categories: 1=yes, I am married, 2=yes, I cohabit with a partner, 3=yes, I do have a partner, but we do not live together, 4=single) |
| Educational attainment | 2.19 | 0.84 | Categorical variable:  1=Low  2=Moderate  3=High | What is the highest level of education you’ve completed?  (7 answer categories: 1=elementary school, 2=lower vocational education, 3=lower general secondary education, 4=intermediate vocational education, 5=upper general secondary education, 6=higher vocational education, 7=university) |
| Wealth | 1.88 | 0.72 | Categorical variable:  1=Low  2=Moderate  3=High | How large do you estimate your total wealth (own house, savings, stocks etc. minus debts/mortgages) to be?  (7 answer categories: 1= < €5,000, 2= €5000 - €25,000, 3= €25,000 - €50,000, 4= €50,000 - €100,000, 5= €100,000 - €250,000, 6= €250,000 - €500,000, 7= > €500,000) |
| Caregiving responsibilities |  |  | Dichotomized variable:  1=Provides care  0=Does not provide care | Do you provide help to family members or friends who are ill or in need of help?  (2 answer categories: 1=yes, 2=no) |
|  |  |  |  |  |
| *Health-related controls* |  |  |  |  |
| Having a chronic health condition | 0.66 | 0.47 | Dichotomized variable  1=Has a chronic health condition  0=Does not have a chronic health condition | Do you have one or more of the following longstanding diseases, as diagnosed by a doctor?  (answer structure: chose between yes or no) |
| *Work-related controls* |  |  |  |  |
| Full-time employment | 0.48 | 0.50 | Dichotomized variable:  1=Employed full-time (>36 hours)  0=Employed part-time (<36 hours) | How many hours per week do you work on average?  (answers are given as number of hours) |
| Supervisory position | 0.25 | 0.44 | Dichotomized variable:  1=In supervisory position  0=In non-supervisory position | Do you have a supervisory position?  (2 answer categories: 1=no, 2=yes) |
| Organizational size | 2.24 | 0.71 | Categorical variable:  1=Small (<50 employees)  2=Medium (50-250 employees)  3=Large (>250 employees) | How many people do approximately work in your work establishment?  (3 answer categories: 1=<50 employees, 2=50-250 employees, 3=>250 employees) |
| Organizational sector | 1.81 | 0.88 | Categorical variable:  1=Government and education sectors  2=Construction sector  3=Health and welfare | Derived through information from the organization |

*Note. SD* = standard deviation, w1 = wave 1, w2 = wave 2
